# The Association Between Blood Coagulation Activity and Lung Function: A Population-Based Study

**DOI:** 10.1371/journal.pone.0015014

**Published:** 2010-11-16

**Authors:** Andrew W. Fogarty, Sarah A. Lewis, Tricia M. McKeever, Gordon D. O. Lowe, Lorna Clark, John Britton

**Affiliations:** 1 Respiratory Biomedical Research Unit, Division of Epidemiology and Public Health, University of Nottingham, Clinical Sciences Building, City Hospital, Nottingham, United Kingdom; 2 Division of Cardiovascular and Medical Sciences, University of Glasgow, Glasgow, United Kingdom; 3 Department of Clinical Chemistry, Nottingham City Hospital, Nottingham, United Kingdom; Leiden University Medical Center, The Netherlands

## Abstract

**Background:**

Increased in susceptibility to thrombotic disease may be associated with lower lung function. If causal, this association may suggest an area for development of new interventions for lung disease. The aim of this study was to investigate the association between blood coagulation activation as measured by plasma d-dimers and lung function.

**Methodology/Principal Findings:**

We conducted a cross-sectional study on 2463 randomly selected adults in 1991 and followed up 1252 of these individuals in 2000. Plasma D-dimer levels, a marker of activity of blood coagulation pathways, were analysed in the baseline 1991 samples. There was an inverse cross-sectional association between plasma D-dimer and Forced Expiratory Volume in one second, with a decrease of 71 ml per µg FEU/ml increment in plasma D-dimer (95% confidence intervals CI: −135 to −6), and a decrease in Forced Vital Capacity (97 ml per µg FEU/ml increase in D-dimer, 95%CI: −170 to −24). These associations were attenuated after adjustment for serum highly sensitive CRP. No association was observed between plasma D-dimer and the decline in lung function between 1991 and 2000.

**Conclusions/Significance:**

The cross-sectional findings are consistent with the hypothesis that activation of blood coagulation pathways is associated with decreased lung function, and that systemic inflammation may contribute to this relation. However, the lack of an association with decline in lung function suggests that clotting pathways that involve d-dimers may not be a promising therapeutic target for new interventions for respiratory disease.

## Introduction

Chronic obstructive pulmonary disease (COPD) is a common disease characterised by reduced lung function and obstructive airflow limitation [1]. Although some exposures, notably smoking [2], pollution [3] and diet [4] are recognised aetiological factors for COPD, residual variation exists between individuals that remains to be explained. Identification of reversible factors demonstrated to modify lung function is a priority, as this may lead to new interventions to prevent chronic lung disease.

Systemic coagulation pathways have the potential to influence pulmonary function as the lungs are a highly vascular organ, and in addition to gas exchange they also have the role of filtering the venous blood, thus preventing thrombotic micro-emboli from entering the arterial circulation. The importance of the lung in filtering the blood of microscopic particles is highlighted in patients with untreated arterio-venous malformations, of whom approximately 36% experience cerebral events, probably as a consequence of micro-emboli in the cerebral circulation [5]. The hypothesis that factors that increase thromboembolic risk may also contribute to structural damage to the lungs is supported by the observation that homozygotes for factor V Leiden gene, who have a genetic predisposition resulting in a hypercoagulable state, had an annual loss of forced expiratory volume in one second (FEV_1_) of 39 ml/year, as compared to 21 ml/year in a study of those who were not homozygotes [6]. Fibrinogen, another constituent of the clotting cascade, is positively associated with an increase in vascular diseases [7], inversely related to lung function cross-sectionally [8], and positively associated with the increased loss of lung function [9] and development of an obstructive lung physiological phenotype [10]. Recent work on the molecular processes that underpin the pathophysiology of COPD has also implicated inappropriate activation of the coagulation cascade and in particular thrombin and plasmin as potential factors that may promote pulmonary inflammation, airway remodelling and eventually the development of emphysema via protease activated receptors [11].

These data thus suggest that higher systemic clotting pathway activity may be a modifiable risk factor for lower lung function. Activation of clotting pathways can be measured by D-dimer, a fibrin degradation product that is a consequence of systemic thrombotic activity [12]. The aim of this study is to test the hypothesis that blood D-dimer levels are associated with lung function using data from a randomly selected population of 2633 individuals based in Nottingham. We have also investigated how these associations may be modified by controlling for levels of C-reactive protein, a marker of systemic inflammation. The results suggest a cross-sectional association between plasma d-dimers and lung function that is attenuated by adjustment for systemic inflammation, but no association between plasma d-dimers and change in lung function over nine years.

## Materials and Methods

### Ethics Statement

The study was approved by the Nottingham City Hospital Ethics Committee.

### Study population

The participants are drawn from a previously reported community-based population of 2633 adults aged 18–70 living in Nottingham first studied in 1991 [13]. In brief, a sample of 7106 adults aged 18 or over were identified from a population of approximately 87 000 living in a Local Authority Area of Nottingham, UK by systematic sampling from a random starting point in the electoral register. All subjects aged 18–70 on 1^st^ January 1991 were invited in writing to attend a local surgery or Health Centre where they were interviewed by a doctor who explained the study protocol. In 2000, all surviving individuals were invited to participate in a follow-up cohort study [4]. Written consent was obtained from all study participants.

### Data collection

In 1991 and 2000 participants were asked to abstain from inhaled bronchodilators for four hours and from oral bronchodilators for eight hours before their study visit. Subjects completed a computer-administered lifestyle questionnaire. FEV_1_ and FVC were then measured using a dry bellows spirometer (Vitalograph, Buckingham, UK), taking the best of three technically satisfactory manoeuvres with the subject seated. After venesection, plasma samples were separated by centrifugation, typically within 15 minutes, and stored at −80° centigrade. These samples were subsequently defrosted and D-dimer measured using an immunoturbidiometric test (Olympus OSR60135) on an Olympus AU5400 analyser (Miami, FL) in 2008.

### Statistical analysis

Smoking status was defined in three categories; current smoker (those who had smoked within one month of the study in 1991 or 2000), ex-smoker (those who had not smoked for at least one month before the appointment in 1991 or 2000) and never smokers. Smoking data from 1991 were used to validate never-smoking status in 2000. The number of pack-years smoking exposure was estimated for subjects from their reported age at starting smoking, and the usual amount smoked during this period.

We analysed the cross-sectional association between plasma D-dimer and FEV_1_ using a similar analytical approach to that used previously in this dataset [13]. We used multiple linear regression with adjustment for *a priori* confounding factors of age, age squared, sex, height, body mass index (BMI), smoking status and total of cigarette pack years smoked. Potential confounding factors including vitamin C and magnesium intake that have been previously associated with lung function were investigated by adding them to the *a priori* model and if the size of effect changed by 10% or more they were considered to be a potential confounding factor and retained in the final model. We examined the shape of the relation between D-dimer and FEV_1_ by plotting D-dimer in quintiles, and linearity was subsequently tested using the likelihood ratio test. As the relation was linear, results were presented as change in FEV_1_ per unit change in plasma D-dimer and also graphically using quintiles to provide a more accessible representation of the size of the associations observed. Repeating the analysis using D-dimer as a logged variable made no appreciable difference to the fit of the model. As serum highly sensitive CRP is positively correlated with D-dimer [14] and also inversely associated with lung function [15], the effect of highly sensitive CRP on the association between D-dimer and lung function was modelled independently as it may be on the causal pathway linking these variables. Interactions for gender, smoking status and BMI on the relation between plasma D-dimer and FEV_1_ and FVC were investigated and considered significant at the value of p<0.05. The analyses were carried out using STATA version ten (Stata Corporation, College Station, Texas).

The longitudinal analysis used both the 1991 and 2000 datasets and the modelling strategy has been previously described [4]. Briefly, predicted FEV_1_ values were modelled in our dataset for each gender in non-smoking, non-asthmatic, non-wheezing individuals with terms for age, height, age-squared and age-height interactions. Individual FEV_1_ values were expressed as the residual difference from the predicted value, and these values were used to create a single value of change in residual by subtracting the 1991 residual from the 2000 residual. The association between plasma D-dimer and change in residual value of FEV_1_ was modelled independently using multiple linear regression to adjust for smoking status, smoking pack years and body mass index.

With over 2400 individuals providing cross-sectional data, and using a standard deviation for FEV_1_ of 900 ml, we calculate that we would have over 90% power to detect a difference of 40 ml in FEV_1_ between quintiles of D-dimer. In the longitudinal analysis, with 1250 samples of plasma, and using data from this dataset of a SD of 291 ml for decline in FEV_1_ over nine years, the study would have over 90% power to detect a linear trend in decline in FEV_1_ with increasing quintiles of D-dimer equivalent to 2 ml/year per increasing quintile.

## Results

2633 individuals provided complete data in 1991 (Table 1) and of these 1346 (51%) were followed up and provided further data in 2000 (Table 2). 2463 (94%) of those who participated in 1991 provided a sample for analysis of plasma D-dimer and these were generally representative of the total study population.

**Table 1:** Baseline characteristics of study population.

	Total study population	Provided blood for baseline D-dimer analysis	Did not provide blood for baseline D-dimer analysis
Number of participants	2633	2463	170
Males N (%)	1312 (50%)	1223 (50%)	89 (52%)
Age mean in yrs (sd)	44.4 (13.6)	44.6 (13.5)	41.3 (14.9)
Mean height m (sd)	1.68 (0.1)	1.68 (0.1)	1.70 (0.1)
Mean body mass index kg/m^2^ (sd)	25.5 (4.0) N = 2614	25.5 (4.0) N = 2445	25.6 (4.5) N = 169
*Smoking status N (%)* Never Ex Curren	1306 (50)	1209 (49)	97 (57)
	730 (28)	694 (28)	36 (21)
	597 (23)	560 (23)	37 (22)
Baseline FEV_1_ - L (sd)	3.19 (0.9)	3.18 (0.9)	3.3 (0.9)
Baseline predicted FEV_1_ % (sd)	99.4 (0.9)	99.6 (17.6)	98.9 (15.9)
Baseline FVC - L (sd)	3.94 (1.1)	3.9 (1.1)	4.0 (1.1)
Baseline Predicted FVC % (sd)	102.9 (16.3)	103.1 (16.3)	100.9 (15.7)
FEV_1_ in 2000 - L (sd)	2.84 (0.85) N = 1343	2.84 (0.85) N = 1275	2.87 (0.78) N = 68
FVC in 2000 – L (sd)	3.67 (1.01) N = 1342	3.67 (1.01) N = 1274	3.67 (0.94) N = 68
Median D-dimer (µg FEU/ml)	-	0.14 (0.08–0.23)	-
Median highly sensitive CRP (mg/l) IQR	1.14 (0.55 to 2.42) N = 2442	1.14 (0.55 to 2.42) N = 2396	1.28 (0.51 to 2.17) N = 46

FEV_1_  =  Forced Expiratory Volume in one second.

FVC  =  Forced Vital Capacity.

IQR  =  interquartile range.

**Table 2:** Rates of follow up and change in lung function of study population by categories of baseline d-dimers in quintiles.

Quintiles of d-dimers in 1991	Number	Provided data in 2000 (%)	Change in FEV_1_ over study period(sd)	Change in FVC over study period (sd)
1	560	288 (51)	−350 (291)	−282 (376)
2	436	218 (50)	−319 (281)	−233 (368)
3	517	264 (51)	−328 (283)	−304 (410)
4	475	266 (56)	−362 (324)	−283 (417)
5	475	242 (51)	−345 (272)	−260 (383)

FEV_1_  =  Forced expiratory volume in one second.

FVC  =  Forced Vital Capacity.

sd  =  standard deviation.

The median value for plasma D-dimer was 0.14 µg fibrinogen equivalent units (FEU)/ml (interquartile range 0.08 to 0.23). In the 1991 dataset serum highly sensitive CRP was positively associated with plasma D-dimer (regression coefficient, +0.012 FEU/ml per increment in highly sensitive CRP in mg/l, p<0.001). Those who attended in both 1991 and 2000 had a median plasma d-dimer of 14 (FEU)/ml, while those who attended in 1991 only also has a median plasma d-dimer of 14 (FEU)/ml (p = 0.44 for difference, Mann-Whitney test).

### Cross-sectional analysis of d-dimers and lung function

**Figure 1:**
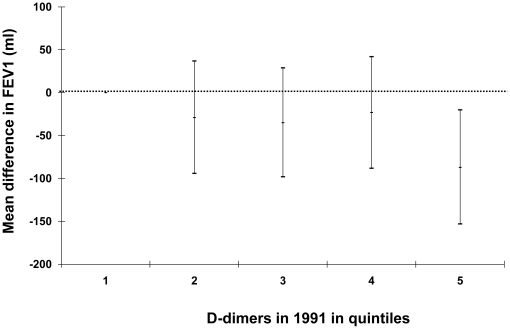
The cross-sectional association of D-dimer (µg FEU/ml) with Forced Expiratory Volume in one second compared to the lowest quintile (reference group). FEV_1_  =  Forced Expiratory Volume in one second adjusted for sex, age, age squared, height, BMI, smoking status (categorical), smoking pack years

There was an inverse association between plasma D-dimer and FEV_1_ (Figure 1), with a decrease of 71 ml per µg FEU/ml increment in plasma D-dimer (95% confidence intervals CI: −135 to −6) after adjusting for potential confounding factors (Table 3). Similar associations were observed between plasma D-dimer and FVC, with a decrease of 97 ml per unit increase in D-dimer (95%CI: −170 to −24). There was no effect modification of any of these associations by smoking status, gender or BMI.

**Table 3:** The cross-sectional association of D-dimer (µg FEU/ml) with Forced Expiratory Volume in one second and Forced Vital Capacity.

	Number of individuals	FEV_1_ (ml)	FVC (ml)
D-dimer*	2445	−71 (−135 to −6)	−97 (−170 to −24)
D-dimer* + highly sensitive CRP	2379	−50 (−115 to +16)	−69 (−143 to +6)

*Model adjusted for sex, age, age squared, height, BMI, smoking status (categorical) and smoking pack years.

95% confidence intervals in brackets.

FEU  =  Fibrinogen equivalent units.

FEV_1_  =  Forced Expiratory Volume in one second.

FVC  =  Forced Vital Capacity.

BMI  =  body mass index.

CRP  =  C-Reactive protein.

Addition of serum highly sensitive CRP to the final model reduced the strength of these associations for both FEV_1_ (−50 ml per µg FEU/ml increase in D-dimer; 95%CI: −115 to +16) and FVC (−69 per µg FEU/ml in D-dimer; 95%CI: −143 to +6). No association was observed between plasma D-dimer and the FEV_1_/FVC ratio in the cross-sectional population.

### Longitudinal analysis of d-dimers and decline in lung function

There was no association between baseline plasma D-dimer and decline in FEV_1_ as measured by the change in residual values over nine years, with a change in residual FEV_1_ of +22.7 ml (95%CI: −27.7 to +73.1) after adjustment for BMI, smoking status and pack years smoked. Similarly, no effect modification of these associations between lung function and plasma D-dimer by smoking status, gender or BMI were observed.

## Discussion

In this study, we report for the first time that higher levels of plasma D-dimer are associated with lower FEV_1_ and FVC cross-sectionally in a randomly selected population and that this association is reduced after adjustment for highly sensitive CRP as a biomarker for systemic inflammation. However, plasma D-dimer levels were not associated with an accelerated decline in lung function over nine years.

The population studied was a random sample from the electoral register of a Local Authority Area in Nottingham, and is thus likely to be a representative sample of the general population. Physiological measurements of lung function and blood samples were collected in a standardised manner. As we used archived serum from 1991 to assess the level of D-dimer that was frozen at −80 degrees Celsius and analysed by one clinical chemist using the same assays and equipment, we are confident that we have minimised bias due to the storage and analysis of the samples. While, we do not have data confirming that the absolute values of d-dimers are the same in long-term frozen samples like ours compared to fresh samples, the standardised storage and analysis of our samples gives us confidence in the integrity of the comparative analyses within the group as a whole. Serum samples stored for similar periods of time have shown significant associations of D-dimer, and CRP, with risk of cardiovascular disease, and with risk factors such as cigarette-smoking [14], [16]. While participation in the follow-up study was potentially biased by survival, nonmigration and motivation to participate, our data suggest that the participants in 2000 were broadly similar to the original population in terms of diet, smoking history, initial lung function and history of respiratory disease [4]. Our response rate of 51% of the original study population raises concerns regarding response bias, particularly as elevated plasma D-dimer is a prognostic marker of increased cardiovascular disease [16] and potentially for mortality, although there was no difference in baseline D-dimer in those who attended for the second visit nine years after baseline compared to those who did not. Thus, it is important to emphasize that our data do not exclude an association between increased activity of systemic coagulation pathways and increased decline in lung function, and that larger population-based studies of change in lung function with shorter intervals between data collection will be required to clarify this important question.

Our original hypothesis was that higher levels of activation of blood coagulation would be associated with lower lung function. Our data were consistent with this hypothesis in the cross-sectional analysis prior to adjustment for systemic inflammation, although the attenuation of this association after adjustment for systemic inflammation suggests that this requires consideration in any further studies of clotting activity and lung function. Our longitudinal analysis demonstrated no association between plasma D-dimer and decline in lung function. We speculate that one potential explanation to reconcile these differing results is that factors in early life that modify lung development also influence activity of the coagulation pathways in later years. This is consistent with the observation that individuals with lower lung function have a higher risk of myocardial infarction [17] – for which elevated D-dimer levels are an independent risk factor [14], [16].

Although the hypothesis that acute changes in blood coaguability in response to particulate pollution may account for some of the variation in cardio-respiratory disease rates is an established one [18], we consider that the concept that chronic differentials in systemic coagulative activity may impact on lung function is an emerging concept supported by Mendelian randomisation techniques. Homozygotes for factor V Leiden gene, who have a genetic predisposition resulting in a chronic hypercoagulable state, have an annual loss of forced expiratory volume in one second (FEV_1_) of almost twice that of non-homozygotes [6]. Similarly, this hypothesis is supported by reports that fibrinogen, another constituent of the clotting cascade, is inversely related to lung function cross-sectionally [8], [10], and also positively associated with increased loss of lung function [9] and the development of obstructive lung physiology [10]. The hypothesis that higher levels of chronic activation of the coagulation pathways may impact on lung pathophysiology is consistent with the observation of the impact of air pollution with regard to the pulmonary vasculature in a study in animals from areas of varying air pollution, demonstrating high levels of microthrombi in the lungs on autopsy in the dogs from Mexico City compared to dogs from less polluted areas where few no micro-thrombi observed [19]. As air pollution is also a risk factor for lower lung function in children [20] it may impact on lung function by a vascular mechanism in addition to its direct toxic effects on the lungs.

Serum highly sensitive CRP has been demonstrated to be inversely associated with lung function in a variety of datasets including ours [15], [21], [22], and as it is indicative of a systemic inflammatory response, we consider that it may be on a causal pathway that links higher coagulation activity with lower lung function. This is consistent with the observation that increased systemic inflammation is associated with elevated blood D-dimer levels both in our dataset and elsewhere [14]. An alternative explanation is that higher serum highly sensitive CRP is a biomarker for lower lung function and that the apparent associations between coagulation pathway activity and lung function that we have observed are in part a consequence of confounding.

Thus, our data are broadly consistent with a range of evidence that increased blood coagulation activity is inversely cross-sectionally associated with pulmonary function, although also demonstrate that future cross-sectional studies in this area should consider systemic inflammation, which may be either a confounding factor or potentially involved in a causal pathway for these associations. However, the lack of an association with decline in lung function suggests that clotting pathways that involve d-dimers may not be a promising therapeutic target for new interventions for respiratory disease.
